# Effect of FAT1 gene expression on the prognosis of medulloblastoma in children: A protocol for systematic review and meta-analysis

**DOI:** 10.1097/MD.0000000000023020

**Published:** 2020-11-13

**Authors:** Jingzhe Yu, Hui Gao, Zeli Su, Feng Yue, Xuanen Tian

**Affiliations:** Department of Pediatric Surgery, General Hospital of Ningxia Medical University, Yinchuan, Ningxia, China.

**Keywords:** children, FAT1, medulloblastoma, meta-analysis, prognosis, protocol

## Abstract

**Background:**

It was reported that cloning human adipose atypical cadherin 1 (FAT1) has an effect on the prognosis of medulloblastoma (MB), while the conclusion still needs to be further proved. Therefore, this study attempted to explore the effect of the high expression of FAT1 on the prognosis of MB children.

**Methods:**

The database was retrieved from China National Knowledge Infrastructure (CNKI), Chinese Biomedical literature Database (CBM), Chinese Scientific and Journal Database (VIP), Wan Fang database, PubMed, and EMBASE. Hazard ratios (HRs) and its 95% confidence intervals (CIs) were applied to assess the prognostic effect of FAT1 on overall survival (OS) and disease-free survival (DFS). RevMan 5.3 and STATA 16.0 software were used to perform the meta-analysis.

**Results:**

The results of the study would be published in peer-reviewed journals or at relevant meetings.

**Conclusion:**

Our findings revealed the effect of the high expression of FAT1 on the prognosis of MB children. Such studies may find a new prognostic marker for MB children and help clinicians and health professionals make clinical decisions.

**OSF Registration Number:**

DOI 10.17605/OSF.IO/5FN8M.

## 1 Introduction

Medulloblastoma (MB) is a highly invasive malignant tumor of posterior cranial fossa, and usually occurs in children.^[1–3]^ It is one of the most common malignant tumors in children, accounting for about 20% of central nervous system tumors.^[4]^ Epidemiological data displays that the annual incidence of MB is about from 0.20 to 0.58 per 100,000 people worldwide.^[5]^ The disease can occur in all age groups, and the peak of diagnosis is between 6 and 8 years old.^[6]^ As a cerebellar embryonal tumor, MB may evolve from primitive neural stem cells. However, the specific pathogenesis is not clear at present. It is the most malignant glioma, because the tumor cells are easy to fall off and spread along the cerebrospinal fluid circulation pathway, with about 30% of patients suffering from metastases at the time of diagnosis.^[7]^

At present, targeted treatment is the first choice of surgery. However, the effect of surgical resection on the prognosis is still inconclusive. Especially, the tumors with severe adhesion to the brainstem are difficult to achieve total resection. It is easy to plant and spread through cerebrospinal fluid, the overall prognosis is poor, the case fatality rate is high, and the 5-year average survival rate is 50% to 75%.^[7–9]^ The latest research illustrated that gene mutation and abnormal intracellular signal transduction are important reasons for the occurrence and development of MB.^[10–12]^ Therefore, the exploration of the key molecules in the occurrence and development of MB is helpful to find new treatment methods, and is of great clinical significance to improve the disease risk stratification, formulate a reasonable treatment plan and evaluate the prognosis of children.

Previous studies indicated that FAT1 is a tumor suppressor gene in gliomas, colon cancer and other tumors,^[13–16]^ and it is important in tumorigenesis and tumor cell proliferation, migration and invasion.^[16]^ However, the high expression of FAT1 in MB children is still controversial. In order to analyze the influence of high expression of FAT1 on childrens MB survival more accurately. This study comprehensively searched literatures on the relationship between the high expression of FAT1 and the prognosis of MB children, and the method of meta-analysis was adopted to evaluate the effect of the high expression of FAT1 on the prognosis of MB children.

## 2 Methods

### 2.1 Study registration

This meta-analysis protocol is based on the Preferred Reporting Items for Systematic Reviews and meta-analysis Protocols (PRISMA-P) statement guidelines. The PRISMA-P checklist for the protocol is provided in the PRISMAP-checklist.

The protocol of the systematic review was registered on Open Science Framework, and the registration number is DOI 10.17605/OSF.IO/5FN8M.

### 2.2 Data sources and search strategy

We searched for China National Knowledge Infrastructure (CNKI), Chinese Biomedical literature Database (CBM), Chinese Scientific and Journal Database (VIP), Wan Fang database, PubMed, EMBASE, and all these electronic databases without language restrictions. The PubMed search strategy was illustrated in detail in Table 1. Other electronic databases applied similar search strategies.

**Table 1:** Search strategy (PubMed).

Number	Search terms
1	Medulloblastoma[MeSH]
2	Medulloblastomas[Title/Abstract]
3	Melanocytic Medulloblastoma[Title/Abstract]
4	Medulloblastoma, Melanocytic[Title/Abstract]
5	Medulloblastomas, Melanocytic[Title/Abstract]
6	Melanocytic Medulloblastomas[Title/Abstract]
7	Medulloblastoma, Childhood[Title/Abstract]
8	Childhood Medulloblastoma[Title/Abstract]
9	Childhood Medulloblastomas[Title/Abstract]
10	Medulloblastomas, Childhood[Title/Abstract]
11	Medullomyoblastoma[Title/Abstract]
12	Medullomyoblastomas[Title/Abstract]
13	Arachnoidal Cerebellar Sarcoma, Circumscribed[Title/Abstract]
14	Sarcoma, Cerebellar, Circumscribed Arachnoidal[Title/Abstract]
15	Medulloblastoma, Desmoplastic[Title/Abstract]
16	Desmoplastic Medulloblastoma[Title/Abstract]
17	Desmoplastic Medulloblastomas[Title/Abstract]
18	Medulloblastomas, Desmoplastic[Title/Abstract]
19	Medulloblastoma, Adult[Title/Abstract]
20	Adult Medulloblastoma[Title/Abstract]
21	Adult Medulloblastomas[Title/Abstract]
22	Medulloblastomas, Adult[Title/Abstract]
23	OR/1-22
24	adipose atypical cadherin 1[Title/Abstract]
25	FAT1[Title/Abstract]
26	OR/24-25
27	Child∗[Title/Abstract]
28	Prognos∗[Title/Abstract]
29	Survival[Title/Abstract]
30	OR/28-29
31	23 AND 26 AND 27 AND 30

### 2.3 Inclusion criteria for study selection

1. According to pathology and histology, patients diagnosed as MB, and under the age of 18
2. FAT1 was expressed in tumor tissues.
3. Reported FAT1 survival-related data, including overall survival (OS) and disease-free survival (DFS)
4. Patients were divided into FAT1 positive and FAT1 negative.
5. Published as full-text articles.

Papers without sufficient data, non-peer-reviewed articles, meta-analyses, literature reviews, case reports, case series, conference summaries, animal studies, letters to the editor, reviews, editorials, and other unrelated studies were excluded from the analysis.

### 2.4 Data collection and analysis

#### 2.4.1 Selection of studies

**Figure 1:**
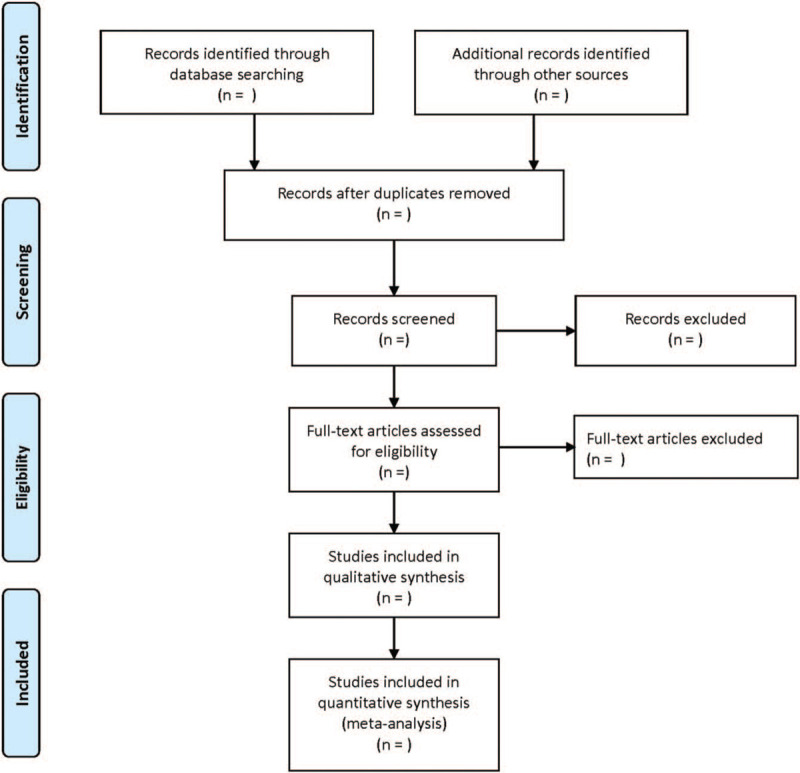
Flow diagram showing literature filtration process.

The titles and abstracts were independently examined by 2 authors to select studies that meet the criteria. In this process, irrelevant literature, reviews, case reports or series, letters, recommendations or guidelines, and studies published only as summaries of meetings were excluded. Then, the full-text level of remaining articles is reviewed by the 2 same reviewers. Differences on the choice of research would be resolved by discussing with the third author. All excluded studies with detailed reasons would be recorded at different stages. The flowchart of the study selection (Fig. 1) could give a detailed description.

#### 2.4.2 Data extraction and management

The 2 authors collected information independently from each included studies. Any conflict was resolved by consensus with the help of a third experienced author. The extracted information included the title of the manuscript, the name of the first author, journal, year of publication, country, race, age, sex, sample size, FAT1 detection method, OS and DFS hazard ratio (HRs) and 95% confidence interval (CIs). We obtained HRs and 95% CIs from the Kaplan–Meier survival curves by referring to Engauge Digitizer version 4.1 (http://digitizer.sourceforge.net/). We contacted the lead authors to obtain any missing or ambiguous information from included studies.

### 2.5 Assessment of quality in included studies

Newcastle-Ottawa quality Assessment scale (NOS), a bias risk assessment tool for observational studies recommended by Cochrane Collaboration, was adopted to evaluate the quality of the included studies.^[17]^ Any dispute was settled through discussion. NOS consists of the following 3 quality parameters: selection, comparability and result evaluation. According to these parameters,^[18]^ each study was scored from 0 to 9.

### 2.6 Measures of prognosis

OS and DFS were taken as prognostic outcomes. The results were expressed as HRs with 95% CIs.

### 2.7 Management of missing data

If there existed insufficient or missing data in the literature, we would only analyze the currently available data and discuss its potential value.

### 2.8 Statistical analysis

Statistical analysis was performed with STATA 14.0 (STATA Corporation, College Station, TX, USA) and RevMan 5.3 (The Nordic Cochrane Centre, The Cochrane Collaboration, 2014). The 95% CIs and HRs were used to evaluate the relationship among FAT1 expression and OS and DFS. Statistical heterogeneity tests were carried out on the included studies. If there was no statistical heterogeneity among the included literatures (*I*^2^ ≤ 50%, *P* < .1), a fixed effect model would be used. When there was statistical heterogeneity among included literatures (*P* < .1, *I*^2^ ≥ 50%), the sources of heterogeneity could be analyzed. Clinical heterogeneity was treated by subgroup analysis. In the absence of significant clinical and methodological heterogeneity, statistical heterogeneity was considered and analyzed by adopting a random effect model. If the clinical heterogeneity of the subgroup analysis wad significantly high, there was only descriptive analysis, rather than meta-analysis.

### 2.9 Additional analysis

#### 2.9.1 Subgroup analysis

According to the detection methods of FAT1, the source of survival data and the high expression threshold of FAT1, we analyzed the subgroup.

#### 2.9.2 Sensitivity analysis

Sensitivity analysis was conducted to assess the impact of individual studies on the overall merger value.

#### 2.9.3 Reporting bias

If the number of studies included in a certain outcome index was no less than 10, funnel chart could be used to evaluate publication bias.^[19,20]^ Besides, Egger and Begg test were adopted for the evaluation of potential publication bias.

### 2.10 Ethics

Our research data was derived from published literature, because there was no patient recruitment and personal information collection. Therefore, ethical approval was not required.

## 3 Discussion

Although advances in surgery, radiotherapy, and chemotherapy improved overall survival, the lifetime sequelae of these treatments was a major health care burden, thus leading to sustained efforts for effective targeted treatments.^[4]^With the development of molecular biology and cytogenetics, it is agreed that gene mutation and abnormal intracellular signal transduction are important reasons for the occurrence and development of MB, and they are closely related to its prognosis.^[21]^ Previous studies proved that MB patients have a series of molecular genetic patterns, such as deletion of tumor suppressor gene PTEN, mutation of p53, and abnormality of SHH and WNT signal transduction pathway.^[22]^ MB contains a variety of molecular subtypes. According to the latest molecular typing study, MB can be divided into 4 subtypes, including WNT type, SHH type, Group3 type and Group4 type. Different molecular subtypes lead to different prognosis, so molecular typing is an important basis for disease risk stratification. However, new and more detailed molecular typing is still in-depth study, and the introduction of signal transduction pathway and molecular typing provides a new idea for individualized treatment and accurate prognosis of MB in children.

At present, it is obvious that FAT1 is abnormally expressed in many kinds of malignant tumors, such as cholangiocarcinoma, breast cancer, oropharyngeal squamous cell carcinoma, diffuse astrocytoma, glioblastoma, acute lymphoblastic leukemia, and so on.^[23]^ RNAi technique was applied to knock down the expression of FAT1 gene in human MB cell line Daoy.^[24]^ It was clear that the proliferation ability of tumor cells is significantly enhanced, suggesting that FAT1 gene may play a role as a tumor suppressor gene in the pathogenesis of MB children. The change of FAT1 gene expression can affect the biological behavior of MB children, after which affect the prognosis of children. Therefore, we would like to conduct a meta-analysis in strict accordance with the PRASMA statement, so as to provide more evidence to support FAT1 as a prognostic marker of MB children. However, this study has some limitations. Our search does not include studies in languages other than Chinese and English, thus may result in certain selective bias. Most importantly, perhaps, there is some heterogeneity due to the application of different treatments in different studies.

## Author contributions

**Conceptualization:** Jingzhe Yu.

**Data curation:** Hui Gao, Zeli Su.

**Funding acquisition:** Jingzhe Yu.

**Resources:** Feng Yue, Xuanen Tian.

**Software:** Feng Yue, Xuanen Tian.

**Supervision:** Jingzhe Yu.

**Writing – original draft:** Jingzhe Yu, Hui Gao.

**Writing – review & editing:** Jingzhe Yu.
